# Comparative Transcriptomic Analysis of the Development of Sepal Morphology in Tomato (*Solanum Lycopersicum* L.)

**DOI:** 10.3390/ijms21165914

**Published:** 2020-08-18

**Authors:** Jingyi Liu, Meijing Shi, Jing Wang, Bo Zhang, Yushun Li, Jin Wang, Ahmed. H. El-Sappah, Yan Liang

**Affiliations:** 1 College of Horticulture, Northwest A&F University, Shaanxi 712100, China; Liujingyi1987@nwsuaf.edu.cn (J.L.); shimeijing4@gmail.com (M.S.); wangjingwj518@gmail.com (J.W.); zhang-bo@nwafu.edu.cn (B.Z.); liyushun2016@nwafu.edu.cn (Y.L.); jw6127@nwafu.edu.cn (J.W.); Ahmed_elsappah2006@yahoo.com (A.H.E.-S.); 2 State Agriculture Ministry Laboratory of Northwest Horticultural Plant Germplasm Resources & Genetic Improvement, Northwest A&F University, Shaanxi 712100, China; 3 Genetics Department, Faculty of Agriculture, Zagazig University, Zagazig 44511, Egypt

**Keywords:** tomato, sepal morphology, RNA-seq, differential expression, cell expansion, auxin, gibberellins, cytokinin

## Abstract

Sepal is an important component of the tomato flower and fruit that typically protects the flower in bud and functions as a support for petals and fruits. Moreover, sepal appearance influences the commercial property of tomato nowadays. However, the phenotype information and development mechanism of the natural variation of sepal morphology in the tomato is still largely unexplored. To study the developmental mechanism and to determine key genes related to downward sepal in the tomato, we compared the transcriptomes of sepals between *downward sepal* (*dsp*) mutation and the wild-type by RNA sequencing and found that the differentially expressed genes were dominantly related to cell expansion, auxin, gibberellins and cytokinin. *dsp* mutation affected cell size and auxin, and gibberellins and cytokinin contents in sepals. The results showed that cell enlargement or abnormal cell expansion in the adaxial part of sepals in *dsp*. As reported, auxin, gibberellins and cytokinin were important factors for cell expansion. Hence, *dsp* mutation regulated cell expansion to control sepal morphology, and auxin, gibberellins and cytokinin may mediate this process. One *ARF* gene and nine *SAUR* genes were dramatically upregulated in the sepal of the *dsp* mutant, whereas seven *AUX/IAA* genes were significantly downregulated in the sepal of *dsp* mutant. Further bioinformatic analyses implied that seven *AUX*/*IAA* genes might function as negative regulators, while one *ARF* gene and nine *SAUR* genes might serve as positive regulators of auxin signal transduction, thereby contributing to cell expansion in *dsp* sepal. Thus, our data suggest that 17 auxin-responsive genes are involved in downward sepal formation in the tomato. This study provides valuable information for dissecting the molecular mechanism of sepal morphology control in the tomato.

## 1. Introduction

The tomato (*Solanum lycopersicum* L.) is an important commercial crop and model for studying the floral organ development of angiosperms. After flowering is completed, tomato sepals are persistently protect young fruits and improve the quality of the appearance of mature fruits. However, as living standards increase, many people started to consider the quality and appearance of tomato sepals. Healthy and flat sepals have become an important standard for measuring the quality of tomato fruits, enhancing visual esthetics and reflecting fruit freshness. Therefore, the molecular mechanism of the regulation of sepal morphology regulation should be investigated.

Sepals affect the flower development by coordinating cell division, cell differentiation and cell expansion with other parts of the flower whorl. The morphology and size of the sepals have been associated with the yield and quality of the fruit. The larger sepal size tightly associates with the protection of flower whorl and better fruit quality [1]. In *SlMBP21*-RNAi tomato, the sepals are longer and fruit sets are improved [2]. Among the green parts of the flower, sepal has the greatest ability to photosynthesis, follow by the receptacle [3]. The contents of Chl and the activity of ribulose-1, 5-bisphosphate carboxylase/oxygenase, the key photosynthetic enzyme, are both increased in longer sepals, and photosynthesis is enhanced in longer sepals, which may contribute by improving the fruit set [2]. Conclusively, sepal morphology is closely associated with fruit development.

Cell size affects sepal morphology. In *Arabidopsis* sepals, the loss of single-cell variability in an *ftsh4* mutant leads to the destruction of the entire sepal shape. This result indicates that changes in individual cell shape and size are important factors influencing the final organ morphology [4]. Other studies have shown that cell growth rate also results in differences in sepal morphology. The growth rate between cells in sepals significantly differs, the relative growth rate of cells in different regions of *Arabidopsis* sepals is about 0% to 5% (cell growth size/h) [5]. Although the cell growth rate during sepal development is variable, all cells achieve the maximum relative growth rate almost at the same time during development [5]. However, cell growth is not synchronized, and the time needed to reach the maximum growth rate varies between cells [5].

The morphological characteristics and growth of plant organs are regulated by hormones, including auxin, cytokinin, gibberellin, ethylene, abscisic acid (ABA), jasmonic acid (JA), brassinosteroids, stearolactone, and many peptides [6,7]. Auxin and gibberellin affect petal expansion and flowering [8,9]. The petal growth of *Arabidopsis thaliana* is regulated by *AUXIN RESPONSE FACTOR8* (*ARF8*), and the petals of *arf8* mutants are significantly larger than those of wild-type (WT) petals because of the increased number of cells and their expansion [8]. The *miRNA319a* mutant in *Arabidopsis* has narrow and short petals, and this trait is regulated by *TCP* transcription factors, which regulate auxin function [10,11,12,13]. A large number of signals (e.g., hormones, mechanical signals and polar fields) are distributed in various regions of plant organs, thereby coordinating the developmental behavior of multiple cells; as a result, different growth regions form. For example, *TCPs* promote the cessation of cell division and slow down the growth rate from the sepal apex to the base, resulting in a mechanical conflict that leads to sepal apex formation. The functional redundancy of these signals reinforces them, thereby enhancing the stability of organ morphology.

Despite a large diversity in flower morphology among *Solanum* species, studies on the genetic basis of the variations in these organs are limited. In the tomato, *MACROCALYX* (*MC*), *TAGL1*, *SlFYFL*, *SlMBP21* and, *SlCMB1*, are involved in the regulation of sepal development [14,15,16,17,18,19]. The T-DNA insertion of *Arlequin* (*Alq*) and the overexpression of the MADS-box gene *TAGL1* result in the conversion of sepals into fleshy, fruitlike organs [15]. The nucleotide sequences of *TAGL1* (syn. *ALQ*) show a high similarity to those of *Arabidopsis* D-class genes *SHATTERPROOF1* (*SHP1* and *AGL1*) [15]. The overexpression of another MADS-box gene, namely *SlFYFL*, gives rise to longer sepals [16]. The MADS-box gene *SlMBP21* regulates the development of sepals and the floral abscission zone [2,17,18]. Moreover, the SEPALLATA (E-function) MADS-box gene *SlCMB1* participates in the development of inflorescence architecture and also regulates sepal size in tomato plants [19].

In this study, high-throughput sequencing was conducted to detect differentially expressed genes (DEGs) during sepal development in the tomato. One wild-type (WT) accession with typical flat-spread sepal and *dsp* mutant with downward sepal were used to identify sepal shape-related candidate genes by comparing sepal transcriptomes at key stages, and to reveal the pathways and related genes possibly involved in sepal development. This work could offer valuable information as a basis for further studying the tomato flower shape. It would also provide insights into the molecular mechanism underlying flower organ development in the tomato for future breeding programs.

## 2. Results

### 2.1. Phenotypic Development Features and Diversity of Tomato Sepals

**Figure 1:**
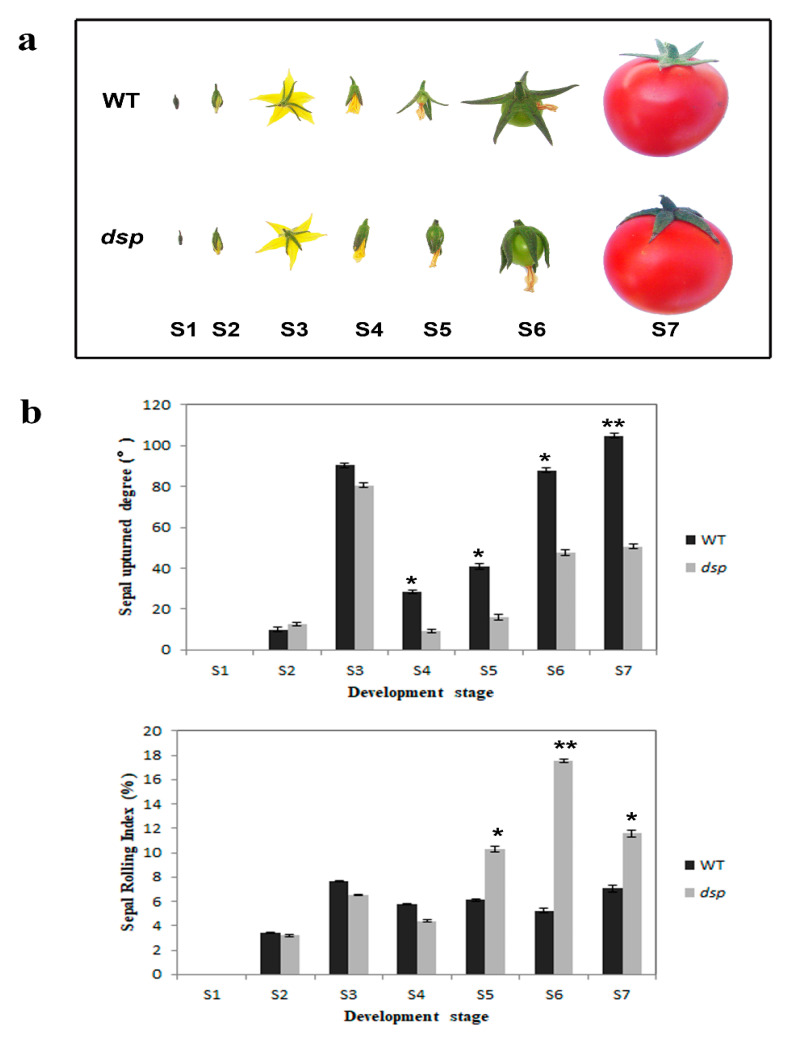
Phenotypes of WT and *dsp (downward sepal)* sepal at different developmental stages. (**a**) Sepal of the wild type and the *dsp* at different stages. (**b**) Sepal upturned degree and sepal rolling index at different stages. *p*-values were determined by *t*-test. * *p* < 0.05; ** *p* < 0.01 (*t*-test).

One sepal morphology mutant was verified. Although the WT tomato plant was characterized by typical flat-spread sepals, the *dsp* mutant, which was generated through natural mutation, exhibited an unusual “downward sepal” phenotype (Figure 1a). The morphological development of sepals from the flower bud stage to the fruit maturity stage in WT and *dsp* was subdivided into seven stages (Figure 1a). At stage 1, i.e., unopened stage, sepals combined with embracing the three inner whorls of floral organs. At stage 2, i.e., slightly opened stage, sepals began to separate, and an inflated corolla emerged at the top of the calyx. At stage 3, i.e., fully opened stage, sepals and petals were completely separated, and the calyx and corolla were fully expanded. At stage 4, i.e., reclosed stage, the calyx and corolla began to close again, and the corolla started to wilt. At stage 5, i.e., reopened stage, the calyx of WT began to open again and bore fruit, but the *dsp* calyx was not completely separated. At stage 6, i.e., morphological differentiation stage, the WT sepals were fully opened again, but the *dsp* sepals were still closed and wrapped around the fruit. At stage 7, i.e., final morphology, the morphological characteristics of sepals were fully developed, the sepals of WT were fully expanded and flat, whereas the *dsp* sepals were oriented downward. Notably, the differences in the sepal morphology between the WT and *dsp* mutant were apparent even at stage 4 of sepal development (Figure 1a,b). The upturned degree and rolling index of the sepals from >10 independent WT and *dsp* plants at different stages were measured. The upturned degree of the sepals of the *dsp* plants was significantly lower than that of the WT plants at stages 4 to 7. The rolling index of the sepals of the *dsp* plants was significantly greater than that of the WT plants at stages 5 to 7 (Figure 1b). The WT and *dsp* plants showed the same morphological differentiation of sepals from stages 1 to 3. Sepal morphology was significantly diverse during stages 4 to 7.

### 2.2. Identification of Differentially Expressed Genes in Sepals from WT and dsp Mutant Plants

**Figure 2:**
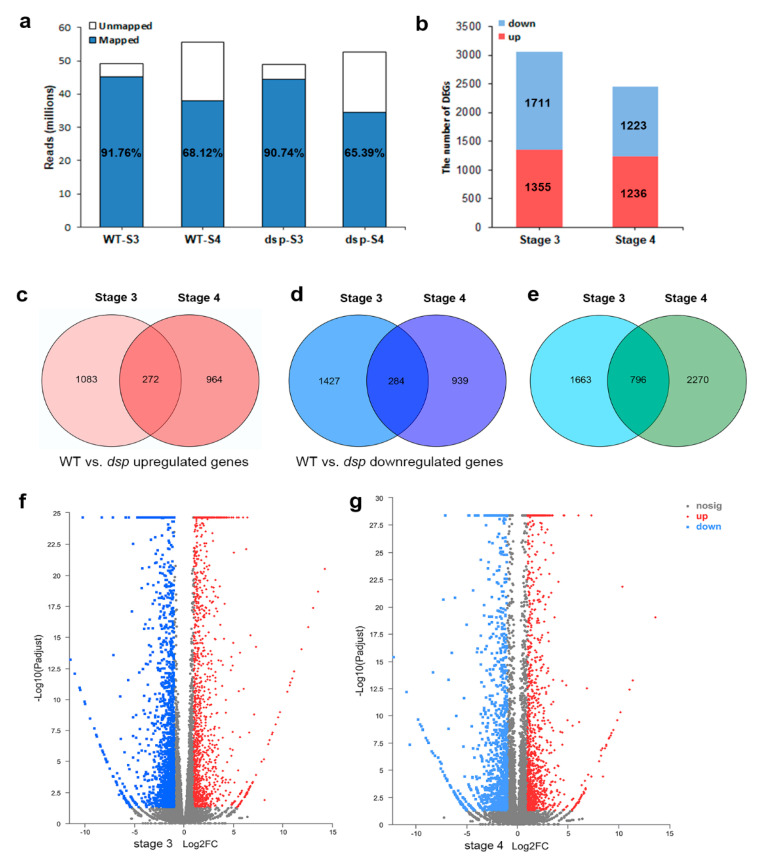
Analysis of transcriptomes from sepals of wild-type and *dsp* plants. (**a**) The number of clean reads obtained from the WT and *dsp* in stage 3 and stage 4, and the percentage of clean reads mapped to the genome. (**b**) The number of up- or down-regulated DEGs for WT vs. *dsp* at stage 3 and stage 4. (**c**) Venn diagram analysis of both upregulated genes of stage 3 and stage 4 groups. (**d**) Venn diagram analysis of both downregulated genes of stage 3 and stage 4 groups. (**e**) Venn diagram analysis of genes with both DEGs of stage 3 and stage 4 groups. (**f**,**g**) The volcano map of differentially expressed genes (DEGs) in stage 3 and stage 4 groups. Red dots indicate upregulated genes; blue dots indicate downregulated genes; grey dots represent no significant difference.

Genome-wide expression analysis was conducted to compare the transcriptome profiles of the sepals between WT and *dsp* mutant plants through a differential gene expression (DGE) approach and to identify the genes involved in the downward sepals of tomato [20]. Phenotypic changes occurred at stage 4 (Figure 1a), so the sepals at stage 3 (0 days after flowering [DAF]; length of ~8 mm) and stage 4 (15 DAF; length of ~11 mm) were chosen for RNA-seq analyses. After the RNA was sequenced, 49.11 million and 48.89 million reads were obtained from the WT and *dsp* plants at stage 3, respectively; of these reads, 91.76% and 90.74% could be mapped to the annotated tomato genome, respectively (Figure 2a). Meanwhile, 55.56 million and 52.56 million reads were obtained from the WT and *dsp* plants at stage 4, respectively. Of these reads, 68.12% and 65.39% could be mapped to the annotated tomato genome, respectively (Figure 2a). The RNA data displayed good correlations between the two biological replicates and were used for further analysis (Figure S1).

After the transcriptomes were compared with those of WT plants, 3066 and 2459 DEGs were detected in the *dsp* plants at stages 3 and 4, respectively, and a total of 4729 DEGs were identified at two stages (Figurer 2b,e). Of the DEGs at stage 3, 1355 were upregulated and 1711 were downregulated (Figure 2b,f). However, 1236 DEGs were upregulated and 1223 DEGs were downregulated in the *dsp* plants at stage 4 (Figure 2b,g).

The top five genes in the upregulated DEGs at stage 3 were two unknown protein-coding genes (*Solyc08g044230.1*, 19065.64-fold; *Solyc05g010380.1*, 3690.21-fold), one wound-induced proteinase inhibitor gene (*Solyc09g084450.2*, 12106.3 9-fold), one 1-aminocyclopropane-1-carboxylate oxidase homolog gene (*Solyc09g089580.2*, 8600.12-fold) and one galactan beta-1,4-galactosyltransferase gene (*Solyc05g015790.1*, 5977.76) (Table S1). The top five significantly downregulated genes at stage 3 were two unknown protein-coding gene (*Solyc01g016460.2*, 2873.56-fold; *Solyc05g032670.1*, 2132.20-fold), one TMV resistance protein (*Solyc07g052790.1*, 2105.26-fold), one phosphoethanolamine N-methyltransferase (*Solyc06g068950.2*, 2083.33-fold) and one BTB/POZ domain-containing protein (*Solyc12g010080.1*, 1515.15-fold). At stage 4, the top five upregulated genes were three unknown protein-coding gene (*Solyc05g010380.1*, 2746.95-fold; *Solyc08g044230.1*, 1315.58-fold, *Solyc05g018060.1*, 1144.65-fold), one 1-aminocyclopropane-1-carboxylate oxidase homolog gene (*Solyc09g089580.2*, 12576.37-fold), and one serine/threonine-protein kinase (*Solyc06g005880.2*, 2158.385-fold). The top five downregulated genes were one ascorbate peroxidase protein (*Solyc06g005150.2*, 4878.05-fold), one unknown protein gene (*Solyc01g016460.2*, 1960.78-fold), one chlorophyll a-b binding protein-coding gene (*Solyc02g071030.1*, 1597.44-fold), one TMV resistance protein-coding gene (*Solyc07g052790.1*, 891.27-fold) and one BTB/POZ domain-containing protein-coding gene (*Solyc12g010080.1*, 780.03-fold). *Solyc09g089580.2* which encodes an ethylene synthesis-related protein, namely, ACO3, was significantly upregulated and remarkably changed in the sepals at both stages.

### 2.3. Annotation of DEGs in Sepals from WT and dsp Mutant Plants

**Figure 3:**
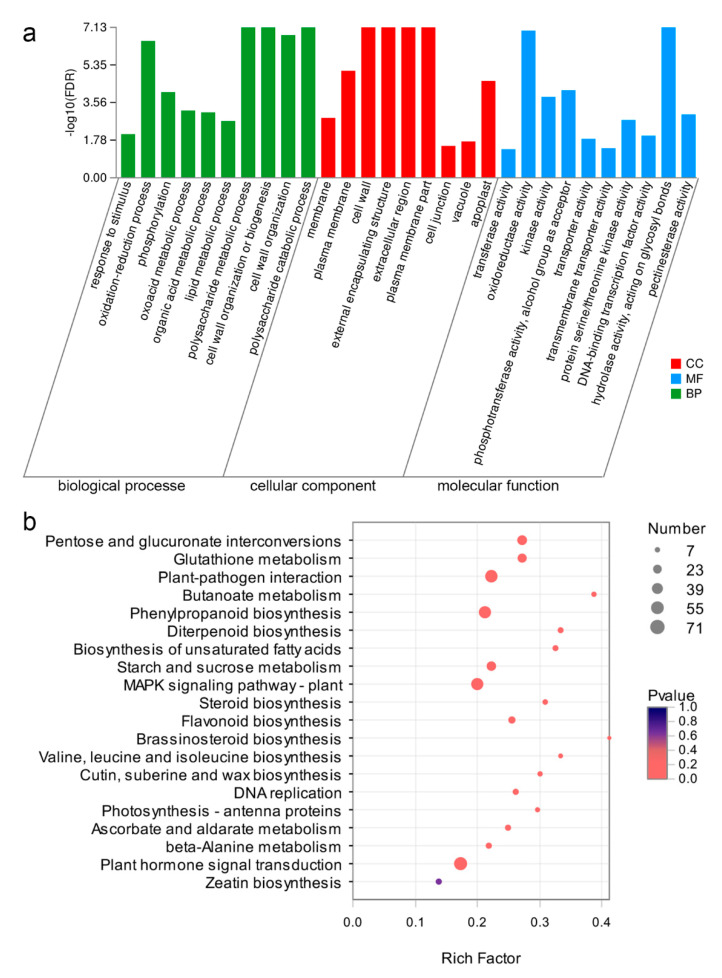
The Gene Ontology (GO) and (Kyoto Encyclopedia of Genes and Genomes) KEGG enrichment analysis of DEGs. (**a**) The GO enrichment analysis. The top ten enriched biological processes, molecular function and cellular component GO terms for DEGs. The x-axis represents GO term. The y-axis represents the significance level of enrichment (−log_10_ FDR—false discovery rate). (**b**) The KEGG enrichment scatter plot of DEGs. The y-axis represents the name of the pathway, and the x-axis represents the rich factor, the degree of KEGG pathway enrichment. Top 20 KEGG pathway enrichments with DEGs were showed. Dot size represents the number of genes and the color indicates the *p*-value.

A total of 4729 DEGs were classified into three categories based on gene ontology (GO) assignments: biological processes (BP), molecular functions (MF), and cellular components (CC). Further GO enrichment analysis resulted in the identification of 205 GO terms (114 BP, 23 CC, and 68 MF; false discovery rate [FDR] <0.05; Table S2). For MF, the top five enriched GO terms were “transferase activity” (738 genes), “oxidoreductase activity” (428 genes), “kinase activity” (307 genes) “phosphotransferase activity” (294 genes), and “transporter activity” (294 genes; Figure 3a; Table S2). For BP, the DEGs were primarily enriched in response to stimuli, lipid metabolism, and organic acid metabolism (Figure 3a; Table S2). Changes in CC occurred primarily in the membrane and cell wall (Figure 3a; Table S2). MF analysis revealed that pectinesterase activity, pectinesterase inhibitor activity, and pectate lyase activity significantly changed, and pectate lyase activity significantly changed according to MF analysis, and they were related to cell wall modification. (Figure 3a; Table S2). The DEGs that function in cell wall organization and biogenesis were enriched. These results suggested that cell wall biogenesis and components in downward sepals were influenced.

Furthermore, 4729 DEGs were mapped into 177 Kyoto Encyclopedia of Genes and Genomes (KEGG) signaling pathways. Of these DEGs, 20 were significantly enriched (*p* < 0.05; Figure 3b; Table S3). The top five enriched KEGG pathways were “brassinosteroid biosynthesis (rich factor = 0.41),” “butanoate metabolism (rich factor = 0.39)”, “taurine and hypotaurine metabolism (rich factor = 0.38)”, “diterpenoid biosynthesis (rich factor = 0.33)”, and “valine, leucine, and isoleucine biosynthesis (rich factor = 0.33),” as shown in Figure 3b and Table S3. KEGG analysis showed that most of the DEGs (71 genes) were enriched in “plant hormone signal transduction”. A number of DEGs were enriched in the following pathways: 15 genes in “diterpenoid biosynthesis,” 7 genes in “brassinosteroid biosynthesis,” and 16 genes in “zeatin biosynthesis”. In addition, many DEGs that were involved in the pathways of cell wall metabolism and “cutin, suberine, and wax biosynthesis” (12 genes) were altered in the *dsp* plants. These results indicated that *dsp* affected sepal morphology by controlling the genes related to cell wall modification and plant hormone regulation.

### 2.4. Coexpression Pattern of DEGs in Sepals from WT and dsp Mutant Plants

**Figure 4:**
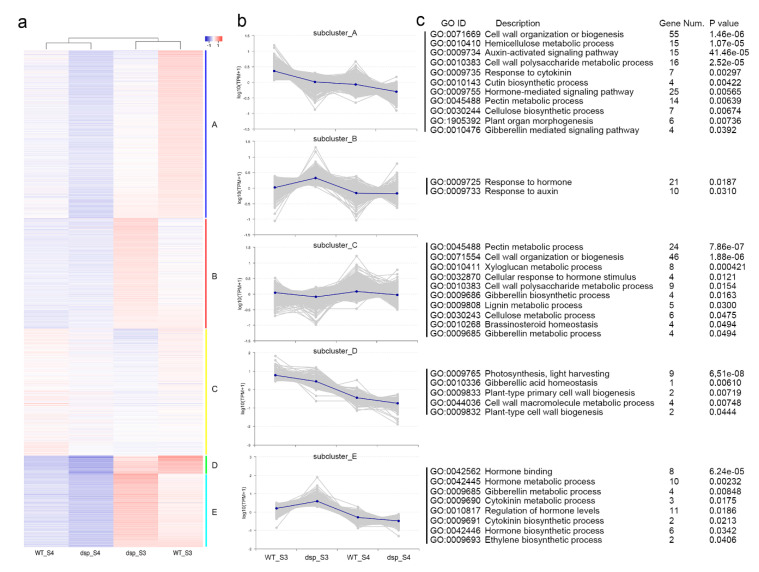
Cluster analysis of differentially expressed genes. (**a**) K-means clustering of DEGs in sepals at stage 3 and 4 of the WT and *dsp* plants. Red and blue in the heat maps represent up-regulated and down-regulated genes, respectively. (**b**) The trend chart of each subcluster. (**c**) The GO enrichment analysis of genes in each cluster.

Hierarchical clustering was applied to 4729 DEGs between WT and *dsp* sepals at stages 3 and 4 to further examine the transcriptomic response to downward sepal. Five large clusters were obtained, and the DEGs were clustered into five clusters by using the *k*-means (*k* = 5) clustering algorithm. These clusters were then visualized with a heat map and a cluster trend chart (Figure 4a,b). The results revealed the general patterns of transcriptomic profiles during sepal development.

The expression levels of the genes in clusters A and C of the *dsp* plants decreased at stage 3, whereas the expression levels of the genes in cluster D of the *dsp* plants continuously decreased from stage 3 to stage 4 (Figure 4b). The expression levels of the genes in cluster C did not significantly differ in the same plant sepals at different stages (Figure 4b). By contrast, the expression levels of the genes from clusters B and E of the *dsp* plants decreased at stage 3. The five clusters were subjected to GO annotation enrichment analysis to gain further insights into the transcriptomic changes in the sepals of the *dsp* plants. In clusters A and C, the genes involved in cell wall organization and biogenesis were enriched (Figure 4c), suggesting that changes in the cell wall were one of the reasons for the altered sepal morphology in the *dsp* plants. Conversely, the gene functioning in hormone metabolism and biogenesis were enriched in clusters B and E. This result indicated that the differences in the sepal morphology of the *dsp* plants were attributed to alterations in hormonal levels, particularly auxin, gibberellin and cytokinin.

### 2.5. Regulation of Cell Expansion by dsp to Control Sepal Morphology

According to the results of DEG analysis, the expression levels of genes related to cell expansion significantly changed in *dsp* sepals (Table 1; Table S4). Among the DEGs, 16 were *XTH* genes, 1 was xyloglucan galactosyltransferase gene, 24 were pectinesterase genes, 1 was pectin methyltransferase gene, 10 were pectin lyase protein genes, 5 were pectin acetylesterase genes, 4 were pectinesterase inhibitor genes, 10 were pectin methylesterase inhibitor genes, 14 were cellulose synthase genes, 1was a glycine-rich cell wall structural protein 1 precursor gene, and 4 were cell wall protein genes; these genes were upregulated or downregulated from 287.36-fold to 2.00-fold (Table 1; Table S4). They were also involved in cell wall metabolism, which is important for cell expansion and cell growth rates. In addition, the expression of 14 expansion (-like) protein genes and 12 extension (-like) protein genes remarkably differed (from 196.85-fold to 2.16-fold; Table 1 and Table S4). These genes were required for cell expansion, cell size, and organ shape development.

**Figure 5:**
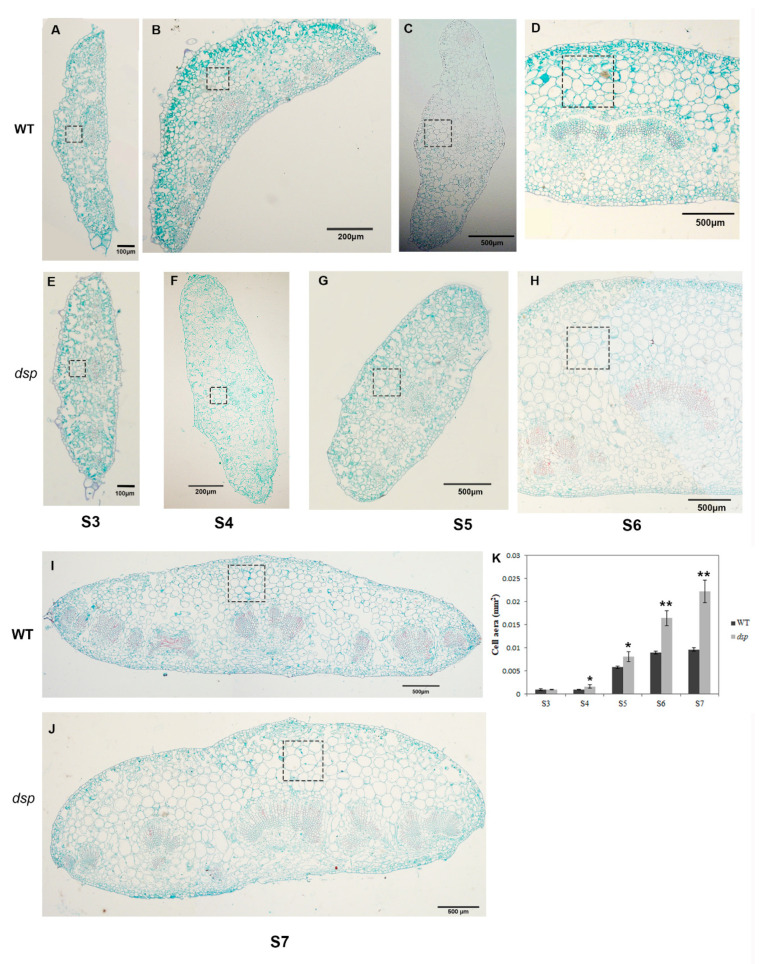
The paraffin cross-section of the sepal of WT and *dsp*. (**A**–**D** and **I**) Stage 3 to 7 of WT sepal. (**E**–**H** and **J**) Stage 3 to 7 of *dsp* sepal. (**K**) The average area of sepal cells in unit view. The area of unit view of A, B, E and F = 0.01mm^2^; C and G = 0.09 mm^2^; D, H–J = 0.25 mm^2^. The cells in > 3 unit views were counted. * *p* < 0.05 (*t*-test), ** *p* < 0.01 (*t*-test).

The cell expansion and cell growth rate determine the size and shape of organs. The DEGs related to cell expansion remarkably varied in *dsp* sepals (Table 1 and Table S4). Hence, the cells in the middle part of the sepal in *dsp* and WT plants were observed through histological analysis. In the sections from stages 4 to 7, the cell size in the adaxial part of the sepals of *dsp* plants was larger than that in the sepals of the WT plants (Figure 5A–K). The cell number per unit view clearly decreased, whereas the cell area significantly increased (*p* < 0.05) in the sepals of *dsp* plants at stages 4 and 5 (Figure 5B,C,F,G,K). The cell area in the sepals of the *dsp* plants at stages 6 and 7 significantly increased (*p* < 0.01; Figure 5D,H–K). Thus, cell enlargement or abnormal cell expansion in the adaxial part of sepals might contribute to downward sepals in *dsp* plants.

The cell expansion and cell growth rate determine the size and shape of organs. The DEGs related to cell expansion remarkably varied in *dsp* sepals (Table 1; Table S4). Hence, the cells in the middle part of the sepal in *dsp* and WT plants were observed through histological analysis. In the sections from stages 4 to 7, the cell size in the adaxial part of the sepals of *dsp* plants was larger than that in the sepals of the WT plants (Figure 5A–K). The cell number per unit view clearly decreased, whereas the cell area significantly increased (*p* < 0.05) in the sepals of *dsp* plants at stages 4 and 5 (Figure 5B,C,F,G,K). The cell area in the sepals of the *dsp* plants at stages 6 and 7 significantly increased (*p* < 0.01; Figure 5D,H–K). Thus, cell enlargement or abnormal cell expansion in the adaxial part of sepals might contribute to downward sepals in *dsp* plants.

Consequently, the alteration of the expression of these cell expansion-related genes induced cell enlargement, which might contribute to downward sepals in *dsp* plants. Therefore, *dsp* might regulate cell expansion to control sepal morphology.

### 2.6. Effects of dsp on Auxin, Cytokinin, and Gibberellin Levels in Sepals

**Figure 6:**
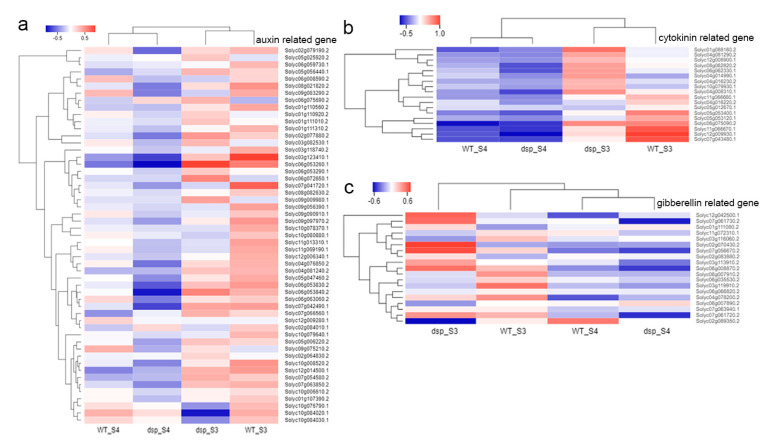
The result of clustering analysis for the differentially expressed genes (DEGs). (**a**) Auxin related genes. (**b**) Cytokinin related genes. (**c**) Gibberellin related genes. Blue and red colors indicate genes with higher expression and lower expression, respectively.

Hormones serve as crucial regulators of organ morphology development. The duration and rate of cell proliferation are positively controlled by auxin and cytokinin. The transition from cell division to expansion is correlated with gibberellin. Phytohormones play a critical role in integrating developmental signals to control organ morphology. The DEGs related to auxin, cytokinin, and gibberellin remarkably differed in *dsp* sepals (Figure 6; Table 1). The majority of these genes were related to auxin, and some of them were associated with cytokinin and gibberellin (Figure 6).

**Figure 7:**
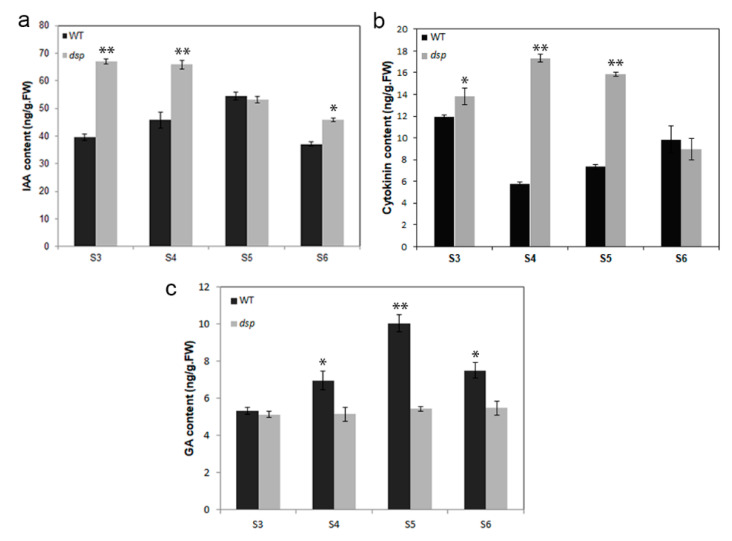
Analysis of hormones in sepals. (**a**) IAA (indole-3-acetic acid) content of sepals in the wild type and *dsp* from stage 3 to stage 6. (**b**) Cytokinin content of sepals in the wild type and *dsp* from stage 3 to stage 6. (**c**) Gibberellin content of sepals in the wild type and *dsp* from stage 3 to stage 6. * *p* <0.05, ** *p*< 0.01 (*t*-test).

Among DEGs, 53 were linked to auxin, i.e., 13 of them were upregulated and 23 were downregulated at stage 3. At stage 4, 9 were upregulated and 16 were downregulated (Figure 6; Table 1; Table S4). The expression levels of the following genes were altered from 404.79-fold to 2.04-fold: 31 auxin-responsive protein-coding genes (*GH3* genes, *SAUR* genes, *ARF* genes, *IAA3*, etc.), four auxin efflux carrier protein-coding genes, nine auxin-induced protein-coding genes, three auxin transporter-like protein-coding genes, two auxin-binding protein-coding genes (ABP19a and ABP19a-like), one auxin-repressed protein-coding genes, one auxin-regulated protein-coding gene, and one small auxin-up protein 58 gene (Figure 6; Table S4). In addition, the content of IAA increased 1.69-fold and 1.44-fold in the *dsp* sepals at stages 3 and 4, respectively, compared with that in the WT sepals (Figure 7a). (Figure 7a). Consequently, *dsp* influenced the efflux, signaling, and content of auxin in sepals.

Among DEGs, 17 were cytokinin synthesis-related factors, and two were cytokinin degradation-related factors. Of these genes, nine were upregulated and nine were downregulated at stage 3; at stage 4, two were upregulated and one was downregulated (Figure 6; Table 1 and Table S4). Two cytokinin riboside 5”-monophosphate phosphoribohydrolase (*SlLOG*) genes were upregulated from 4.01-fold to 2.94-fold at stage 3, one isopentenyltransferase 2 (*SlIPT*) gene was downregulated up to 5.39-fold, and one *LOG* gene was upregulated up to 3.06-fold at stage 4. Two cytokinin oxidase/dehydrogenase (*SlCKX*) genes were upregulated from 6.54-fold to 3.33-fold at stages 3 and 4 (Table 1, Figure 6). The first step of CK biosynthesis in plants is the N-prenylation of adenosine 5ʹ-phosphates via dimethylallyl diphosphate, resulting in the biosynthesis of CK nucleotides. [21,22]. This step is catalyzed by IPT. The next step is the phosphoribohydroxylation of CK nucleotides to synthesize biologically active CK nucleobases [23]. This step is catalyzed by CK riboside 5′-monophosphate phosphoribohydrolase (LOG). Cytokinin conjugation occurs mainly through cytokinin oxidase (CKX) enzymes. Furthermore, two zeatin O-glucosyltransferase genes were upregulated from 4.75-fold to 4.14-fold, whereas four zeatin O-glucosyltransferase genes were downregulated from 5.01-fold to 2.03-fold) (Table 1 and Table S4). Three zeatin O-xylosyltransferase genes were upregulated from 11.02-fold to 3.17-fold, whereas four zeatin O-xylosyltransferase genes were downregulated from 5.96-fold to 2.64-fold) (Table 1 and Table S4). Cytokinins with a hydroxyl group on the side chain can undergo O-glycosylations. Zeatin O-glucosyltransferase and zeatin O-xylosyltransferase, which are *zisZOG* genes, play important roles in regulating the levels of *cis*-zeatin and maintaining appropriate levels of active cytokinins because biosynthetic enzymes work slowly [24]. Zeatin O-glycosyl derivatives are resistant to the cytokinin degrading enzyme *CKX* and are considered to be storage forms because they can be cleaved by b-glucosidase [22,25]. In our study, the contents of cytokinin in the *dsp* sepals increased 1.16-, 2.35- and 1.34-fold at stages 3, 4, and 5, respectively, compared with those in the WT sepals (Figure 7). In our data, the upregulation of *SlCKX* might be responsible for the increase in the cytokinin levels in the sepals of *dsp* plants. The upregulation of *SlLOG* might accelerate cytokinin synthesis and result in an increase in cytokinin levels. These results indicated that the increased expression of cytokinin biosynthesis-related genes (*SlLOGs*), the increased expression of *SlCKXs*, and the increased cytokinin content contributed to the expansion of sepal cells in *dsp*.

GAs are essential for cell elongation and other plant growth and developmental processes [26,27,28]. GA20oxs (GA20-oxidases) are key GA biosynthesis regulators that determine the GA content of plant species [29]. Among the DEGs in our study, seven were gibberellin synthesis-related genes and 11 were gibberellin response-related genes. Of these genes, eight were upregulated and seven were downregulated at stage 3 while six were upregulated and three were downregulated at stage 4 (Figure 6; Table 1 and Table S4). The two GA20ox genes in *dsp* were downregulated from 10.87-fold to 4.31-fold at stage 3. One of them was downregulated to 2.92-fold at stage 4. Two GA2ox genes, which are responsible for the deactivation of GAs, were significantly upregulated from 4.98-fold to 3.81-fold at stage 3 of the *dsp* plant (Table 1 and Table S4). Furthermore, the contents of gibberellin in the *dsp* sepals of the plant decreased by 1.35- and 1.85-fold at stages 4 and 5, respectively, compared with those in the WT sepals (Figure 7). These results indicated that the decreased expression of GA biosynthesis-related genes (GA20oxs) and the increased expression of GA2oxs contributed to a decrease in the GA content.

### 2.7. Possible Function of 17 Auxin-Responsive Genes as Regulators in Downward Sepal Formation in the Tomato

**Figure 8:**
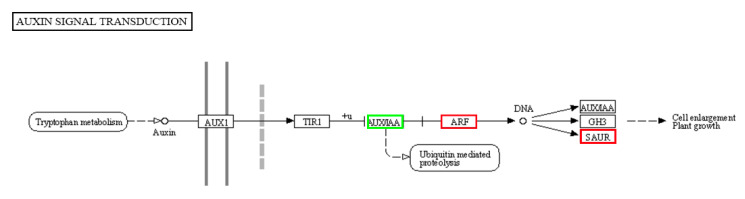
KEGG graph of auxin signal transduction pathway. Up-regulated, down-regulated and unchanged genes are shown in red, green and black boxes, respectively. “ARF” in the red box indicates the one *ARF* gene (*Solyc04g081240.2*). “AUX/IAA” in the green box represents the seven *AUX/IAA* genes (*Solyc06g008590.2*, *Solyc08g021820.2*, *Solyc09g083290.2*, *Solyc09g090910.1*, *Solyc04g076850.2*, *Solyc06g053830.2*, *Solyc06g053840.2*). “SAUR“ in the red box indicates the nine *SAUR* genes (*Solyc05g025920.2*, *Solyc05g056440.1*, *Solyc01g110920.2*, *Solyc03g082530.1*, *Solyc06g053290.1*, *Solyc06g072650.1*, *Solyc09g009980.1*, *Solyc07g066560.1*, *Solyc02g084010.1*).

The detected DEGs were mapped to reference canonical pathways in the KEGG to further identify the key gene regulatory pathways responsible for the formation of downward sepals in the tomato [30]. Among the enriched KEGG pathways, the “auxin signal transduction” pathway, which was putatively associated with the downward sepal phenotype in the *dsp* mutant, was also enriched. Notably, one *ARF* gene (*Solyc04g081240.2*) and nine *SAUR* genes (*Solyc05g025920.2*, *Solyc05g056440.1*, *Solyc01g110920.2*, *Solyc03g082530.1*, *Solyc06g053290.1*, *Solyc06g072650.1*, *Solyc09g009980.1*, *Solyc07g066560.1*, *Solyc02g084010.1*) were dramatically upregulated in the *dsp* sepals of *dsp* mutant, whereas seven *AUX/IAA* genes (*Solyc06g008590.2*, *Solyc08g021820.2*, *Solyc09g083290.2*, *Solyc09g090910.1*, *Solyc04g076850.2*, *Solyc06g053830.2*, *Solyc06g053840.2*) were significantly downregulated in the *dsp* sepal mutant (Table 1 and Table S4). All of them were mapped to the “auxin signal transduction”. Auxin signaling promotes cell expansion. In this pathway, once the concentration of auxin increases, it mediates the linkage of TIR1/AFBs with AUX/IAAs, and AUX /IAA proteins are degraded by proteasomes [31,32,33]. AUX /IAA proteins are repressors of auxin response factors (*ARFs*) that function as activators, and their degradation leads to the activation of the transcriptional regulation of *ARFs* (Figure 8; Table S4). Auxin induces cell expansion through the degradation of AUX/IAAs and the activation of *ARFs*. *ARFs* are transcription factors that bind to the promoters of auxin-responsive genes [31,34,35,36]. In Arabidopsis, *ARF7* positively regulates the expression of *EXP8* [37], which participates in extensive cell growth [38]. In addition, 9 *SAUR* genes stimulated the activity of H+-ATPase proton pumps in the plasma membrane to promote cell expansion (Figure 8) [39]. These observations indicated that the seven AUX/IAA genes might function as negative regulators, while one *ARF* gene and nine *SAUR* genes might serve as positive regulators of auxin signal transduction. These genes could play a positive role in cell expansion and the downward morphology of the sepals of *dsp* mutant plants.

## 3. Discussion

The histological analysis showed that adaxial cells in the *dsp* sepals were larger than those in the WT sepals (Figure 6). Cell expansion is an important developmental force of organ morphology development at the cellular level. The transcriptome data indicated that cell expansion-related genes, such as *XTH*, pectinesterase genes, pectin lyase, cellulose synthases, expansins, and extensins, significantly changed (Table 1 and Table S4). Plant cell walls are complex structures composed of cellulose, xyloglucan, pectic polysaccharides, and structural proteins. To adapt to expansive forces and plant growth, plant cells selectively loosen their cell wall [40]. In the *dsp* sepals, seven *XTH* genes were upregulated at stage 4, and these genes were responsible for loosening the cell wall and promoting cell expansion and floral organ growth during flower opening [41,42]. Xyloglucan endotransglycosylase/hydrolases (XTHs) are encoded by a large multigene family, which cut xyloglucan and join the new reducing end to the non-reducing end of another xyloglucan (a transglucosylation) or to water (a hydrolysis) [43]. XTH itself cannot induce wall relaxation or creep, but can synergistically enhance wall extension, which might be termed an indirect or secondary loosening agent [43]. Moreover, 34 genes related to cell wall degradation were expressed differently in *dsp* sepals. For example, pectate lyase and pectinesterase break down pectins and participate in cell wall metabolism [44,45,46]. Ten pectin methylesterase (PME) inhibitor genes were expressed differently in *dsp* sepals, and they are implicated in the demethylation of pectin, resulting in the relaxation of the cell wall so that cells can enlarge [47]. Twelve DEGs related to cellulose synthesis were downregulated at stages 3 and 4 and involved in depositing cellulose to either primary or secondary walls in *Arabidopsis* [48]. In addition, 26 expansin/expansin-like and extensin/extensin- like genes were expressed differently in *dsp* sepals. They were considered two of the most important regulators of cell wall expansion and loosening during plant cell growth [49,50,51]. The α-expansins induced wall creep and wall relaxation, which mediated acid-induced extension of plant cell walls without mechanically weakening the cell walls [52,53,54]. In our study, 16 *XTHs*, 14 expansin genes and 11 PMEs/PME inhibitor genes were expressed differently in *dsp* sepals (Table S4). These results suggested that *dsp* positively regulated cell expansion through cell wall loosening, thus inducing the alteration of sepal morphology.

Organ development is regulated by different phytohormones, which manipulate appropriate cell division and cell expansion. As an important hormone in plant development, auxin plays important role in cell expansion [55,56,57,58]. In our work, the IAA content increased in *dsp* sepals, compared with those in WT sepals (Figure 7A). Moreover, the expression of auxin-related genes involved in response and efflux, especially *AUX/IAA* and *SAUR* family genes, significantly varied (Figure 8; Table S4). *SMALL AUXIN UP-RNA* (*SAUR*) genes constitute the largest family of auxin-induced genes, and 79 members are found in *Arabidopsis* [34]. The overexpression of stabilized SAUR19 proteins confers numerous phenotypes indicative of increased cell expansion, including increases in hypocotyl length and leaf size, and altered tropic growth [59,60,61] Similar findings were obtained with plants expressing SAUR63 proteins [62], suggesting that SAURs may positive effectors of cell expansion [59,62,63]. SAUR gene expression is upregulated by treatments/conditions that promote growth (e.g., IAA, brassinosteroids) and downregulated by factors that repress growth (e.g., abscisic acid, jasmonic acid, abiotic stress) [64,65]. Auxin is known to stimulate the activity of plasma membrane H^+^-ATPase proton pumps, which promote proton efflux to acidify the apoplast and facilitate the uptake of solutes and water to drive plant cell expansion [39,66,67,68,69,70,71]. This process is regulated by auxin-inducible SAUR proteins [39,70]. The ensuing decrease in apoplastic pH alters the activity of cell wall-modifying proteins, including expansins [72], xyloglucan endotransglycosylase/hydrolases (XTHs) [73], and pectin methylesterases (PMEs) [74], leading to changes in wall extensibility. Auxin promotes the reorientation of microtubules from random to transverse, and suppresses the peroxidase activity in the cell wall. Consequently, cell wall extensibility is also promoted [75]. Therefore, *dsp* likely affected cell expansion by regulating auxin response, which induced the alteration of sepal morphology.

Transcriptomic analysis demonstrated that the genes related to cytokinin were expressed differentially; correspondingly, the cytokinin production level clearly increased in the *dsp* sepals compared with that in the WT sepals (Figure 7B). Furthermore, 19 DEGs were related to cytokinin: 17 biosynthesis-related genes (11.02-fold, the maximum fold change) and two degradation-related genes (6.54-fold, the maximum fold change; Figure 6C; Table S4). For example, two cytokinin oxidase/dehydrogenase (*CKX*) genes were upregulated 2.71-fold to 1.74-fold (Table S4). The overexpression of *CKX1* or *CKX2* in *Arabidopsis* and other species causes the elongation of the primary root and increases root branching [76,77,78], whereas the overexpression of *AtCKX7* results in an opposite phenotype [79]. Therefore, each *CKX* determined a specific developmental and physiological function. Cytokinin stimulated cell expansion in plants [80,81] and induced fourα-expansin subfamily members, namely, *EXPA1*, *EXPA10*, *EXPA14*, and *EXPA15*, in Arabidopsis root [82]. As a result, the cell wall loosened, and the cells expanded. In angiosperms, the ancient *tRNA-IPTs* and *CKXs* preferred the cZtype cytokinins as substrates, play a housekeeping role to maintain basic cellular functions. On the other hand, the nonancient ATP/ADP-IPTs and CKXs preferred the iP- and tZ-type cytokinins as bstrates, contribute more to the regulation of organ development and abiotic stress responses [83]. Five zeatin O-glucosyltransferase and zeatin O-xylosyltransferase (cisZOG) genes were upregulated 3.46 fold to 1.67-fold. cisZOG is involved in zeatin biosynthesis [24]. These results implied that cytokinin biosynthesis and signaling in *dsp* sepals were affected, and these processes might promote cell expansion and lead to downward sepals.

Gibberellin promotes cell expansion [84]. In *Petunia* and *Mimulus*, stamen removal leads to a reduced number of petals likely because stamens produce gibberellins [85,86]. In addition, GA-deficient *Arabidopsis* mutants exhibit a reduction in petal elongation, but gibberellins promote cell elongation by inhibiting the function of DELLA protein. The DELLA protein RGA, RGL1 and RGL2 in *Arabidopsis* inhibit the growth of petals in gibberellin-deficient plants [87]. Our results revealed that the content of gibberellins decreased, whereas the cell size increased in the *dsp* sepals, suggesting that gibberellins antagonized CK in a wide range of developmental events, including cell differentiation, shoot and root elongation, and meristem maintenance [88,89]. Active CK and recessive GA signals not only induce the expression of GA2ox, a GA-deactivating enzyme but also promote the expression of the cytokinin-biosynthesis gene *ISOPENTENYL TRANSFERASE7*. Consequently, cytokinin signaling is elevated [89,90]. Therefore, *dsp* promoted downward sepals through the regulation of adaxial cell size in sepals, and this process might be mediated by auxin, cytokinin and gibberellin.

## 4. Materials and Methods

### 4.1. Plant Materials

The *dsp* mutant of tomato was generated in the background of the inbred line TI1101 through natural mutation and stabilized via six generations of selfing prior to this study. The seeds of *dsp* mutant and WT were germinated on wet filter paper in a Petri dish at 28 °C in dark for 3 days. Then the resulting seedlings were grown in a growth chamber under a 16h/8h (light/dark) photoperiod with 25 °C/16 °C temperatures, respectively. Upon four true-leaf stage, plants were transferred to a greenhouse in the experimental field of the Northwest A&F University. Pest control and water management were carried out according to standard practices. All the materials were grown in a plastic greenhouse in the Northwest A&F University (Shaanxi, China).

### 4.2. Measurement of Sepal Morphology at Different Developmental Stages

**Figure 9:**
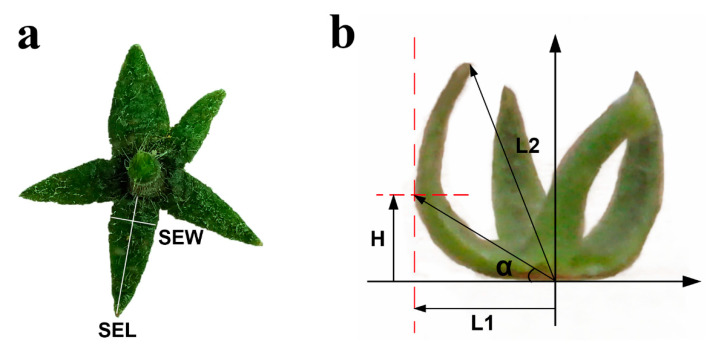
Measurement of sepal traits. (**a**) Measurement of sepal length (SEL) and sepal width (SEW). (**b**) Measurement of Sepal Upturned Degree (SEUD) and Sepal Rolling Index (SERI). SEUD-Sepal Upturned Degree (α). α = arctan (H/L1) (°). H represents the vertical distance between the furthest point from sepal to stalk in the horizontal line and the base line; L1 is the longest distance from sepal to stalk. SERI-Sepal Rolling Index = (SEL − L2)/SEL. SEL, Sepal Length; L2 is the distance from the sepal apex to base point.

Nine ripened fruits with whole calyx per accession were used as repeats in the phenotypic analysis. Each sepal was evaluated for 2 traits:

(1) Sepal Upturned Degree (SEUD, °) = 90° + α = 90° + arctan (H/L_1_), degree of upwarp or down-wrap of sepal. Put a whole calyx on a L-square ruler, then keep the calyx base level on one side of L-square ruler and the sepal to be measured leaning against another side of L-square ruler. H represents the vertical distance between the furthest point from sepal to stalk in the horizontal line and the base line, L_1_ is the longest distance from sepal to stalk (Figure 9).
(2) Sepal Rolling Index (SERI, %) = (SEL − L_2_)/SEL × 100%, SEL (Sepal Length, mm) is the length of sepal in flat condition, L_2_ is the distance from apex to base point (Figure 9).

### 4.3. DGE (Differentially Gene Expression) Library Construction and Sequencing

Sepal at stage 3 (0 DAF, length around 8mm) and stage 4 (15 DAF, length around 11 mm) were collected from WT and *dsp* mutant at the same time on the same day. Samples were immediately frozen in liquid nitrogen and stored at −80°C for RNA-Seq analyses. Total RNA was isolated using the TRIzol^®^ Plant RNA Purification Reagent according to the manufacturer’s instructions (Invitrogen, Carlsbad, CA, USA) and genomic DNA was removed using DNase I (TaKara, Dalian, China). RNA quality was determined by 2100 Bioanalyser (Agilent, Silicon Valley, CA, USA) and quantified using the ND-2000 (NanoDrop Technologies, Wilmington, DE, USA). Only high-quality RNA sample (OD260/280 = 1.8–2.2, OD260/230 ≥ 2.0, RIN ≥ 6.5, 28S:18S ≥ 1.0, >10 μg) was used to construct sequencing library.

RNA-seq transcriptome library was prepared following TruSeq^TM^ RNA sample preparation Kit from Illumina (San Diego, CA, USA) using 5 μg of total RNA. Shortly, messenger RNA was isolated according to polyA selection method by oligo(dT) beads and then fragmented by fragmentation buffer firstly. Secondly double-stranded cDNA was synthesized using a SuperScript double-stranded cDNA synthesis kit (Invitrogen, Carlsbad, CA, USA) with random hexamer primers (Illumina, San Diego, CA, USA). Then the synthesized cDNA was subjected to end-repair, phosphorylation and “A” base addition according to Illumina’s library construction protocol. Libraries were size selected for cDNA target fragments of 200–300 bp on 2% Low Range Ultra Agarose followed by PCR amplified using Phusion DNA polymerase (NEB) for 15 PCR cycles. After quantified by TBS380, the paired-end RNA-seq sequencing library was sequenced with the Illumina HiSeq xten (2 × 150 bp read length).

The raw paired end reads were trimmed and quality controlled by SeqPrep (https://github.com/jstjohn/SeqPrep) and Sickle (https://github.com/najoshi/sickle) with default parameters. Then clean reads were separately aligned to reference genome with orientation mode using TopHat (http://tophat.cbcb.umd.edu/,version2.0.0) [91] software. The mapping criteria of bowtie was as follows: sequencing reads should be uniquely matched to the genome allowing up to 2 mismatches, without insertions or deletions. Then, the region of the gene was expanded following depths of sites and the operon was obtained. In addition, the whole genome was split into multiple 15k bp windows that share 5k bp. New transcribed regions were defined as more than 2 consecutive windows without the overlapped region of the gene, where at least 2 reads mapped per window in the same orientation.

### 4.4. Bioinformatics Analysis of DGE Data

To identify DEGs (differential expression genes) between two different samples, the expression level of each transcript was calculated according to the fragments per kilobase of exon per million mapped reads (FRKM) method. RSEM (http://deweylab.biostat.wisc.edu/rsem/) [92] was used to quantify gene abundances. R statistical package software EdgeR (Empirical analysis of Digital Gene Expression in R, http://www.bioconductor.org/packages/2.12/bioc/html/edgeR.html) [93] was utilized for differential expression analysis. In addition, functional-enrichment analysis including GO and KEGG were performed to identify which DEGs were significantly enriched in GO terms and metabolic pathways at Bonferroni-corrected *p*-value ≤ 0.05 compared with the whole-transcriptome background. GO functional enrichment and KEGG pathway analysis were carried out by Goatools (https://github.com/tanghaibao/Goatools) and KOBAS (http://kobas.cbi.pku.edu.cn/home.do) [94].

### 4.5. Paraffin Sectioning and Electron Microscopy Experiment

The sepal cells were observed by paraffin sections stained with safranin-fast green. The sepals of WT and *dsp* at stage 3 to stage 7 were harvested and immediately fixed in formalin/glacial acetic acid (FAA). The samples were then dehydrated in a xylene and alcohol series (75, 85, 90, 95 and 100%) and then embedded in paraffin wax. Four-micrometer-thick sections were cut and stained with safranin (1%)-fast green (0.5%) for histological examination. Sepal cells underwent electron microscopy using a general laboratory biology electron microscope

### 4.6. Extraction, Purification and Quantification of the Phytohormones in Sepals

Stage 3, stage 4, stage 5 and, stage 6 sepals for phytohormones IAA, zeatin and GA quantification. The method for extraction, purification, and quantification of phytohormones was modified from the description of Wang [95]. ELISA kits used for estimation of the hormonal levels came from China Agricultural University (Beijing, China).

SPSS software was used for statistical analysis. The *dsp* mutants were compared with the WT using Tukey’s test at *p* < 0.05.

## 5. Conclusions

Conclusively, in the first part of the study, we observed the seven stages of the sepal morphological development of *dsp* (downward sepal) mutant and WT. The WT and *dsp* plants showed to be significantly diverse during stages 4 to 7. To understand the molecular mechanisms of downward sepal development, a transcriptome analysis of *dsp* mutant and WT plants at stages 3 and 4 was performed. We observed that *dsp* affected sepal morphology by controlling genes related to cell wall biogenesis and modification and plant hormone regulation, particularly auxin, gibberellin, and cytokinin. Among these genes, seven AUX/IAA genes functioned as negative regulators, while one *ARF* gene and nine *SAUR* genes served as positive regulators of auxin signal transduction. According to cell morphology observation between *dsp* and WT, we found that cell enlargement or abnormal cell expansion in the adaxial part of sepals might contribute to downward sepals in *dsp*. Meanwhile, *dsp* mutant led to increase in auxin and cytokinin, and a decrease in gibberellin. In conclusion, *dsp* promoted downward sepals through the regulation of adaxial cell size in sepals, and this process might be mediated by auxin, cytokinin and gibberellin.

## Figures and Tables

**Table 1:** The top 10 DEGs related to cell expansion, auxin, gibberellins and cytokinin in *dsp* sepal at stage 3 and stage 4.

ID	logFC	*p* value	Annotation
Cell expansion (stage 3)			
Solyc02g080220.2	1.61	4.34 × 10^−53^	Pectinesterase
Solyc07g052980.2	−1.30	3.22 × 10^−33^	Xyloglucan endotransglucosylase/hydrolase protein 9
Solyc08g005800.2	−1.71	2.64 × 10^−36^	Pectin acetylesterase
Solyc07g043390.2	−1.79	1.11 × 10^−90^	cellulose synthase-like protein G2
Solyc02g088100.2	−1.99	4.22 × 10^−47^	Expansin-A5
Solyc02g078040.2	−2.16	6.28 × 10^−60^	pistil-specific extensin-like protein
Solyc05g007830.2	−2.39	1.79× 10^−59^	Expansin-A15
Solyc05g014000.2	−2.78	2.60× 10^−180^	Pectate lyase
Solyc03g083770.1	−4.14	0	pectin methylesterase inhibitor
Solyc06g005560.2	−4.60	1.83 × 10^−35^	Expansin9
Cell expansion (stage 4)			
Solyc01g106650.2	3.28	9.68 × 10^−58^	Xyloglucan endotransglucosylase/hydrolase protein 10
Solyc06g034370.1	1.64	6.36 × 10^−134^	pectin methylesterase inhibitor
Solyc03g097050.2	1.46	4.00 × 10^−65^	cellulose synthase-like protein D3
Solyc07g017600.2	1.28	7.32 × 10^−60^	Pectinesterase
Solyc04g074290.2	1.28	2.21 × 10^−74^	pectin methyltransferase QUA2
Solyc03g083730.1	−1.08	1.76 × 10^−59^	pectin methylesterase inhibitor
Solyc03g025600.2	−1.17	4.64 × 10^−62^	Pectin acetylesterase
Solyc09g097770.2	−1.34	1.22 × 10^−168^	Cell wall protein
Solyc08g077330.2	−2.44	2.38× 10^−107^	expansin-like B1
Solyc06g084620.1	−2.86	2.12 × 10^−48^	Pectinesterase
Auxin (stage 3)			
Solyc05g025920.2	8.66	1.98 × 10^−8^	Auxin-induced protein 15A
Solyc06g075690.2	2.23	1.51 × 10^−91^	Auxin-regulated protein
Solyc09g075210.2	1.08	3.11 × 10^−32^	indole-3-acetic acid-induced protein ARG2
Solyc02g077880.2	1.05	1.14 × 10^−36^	Auxin repressed/dormancy associated protein
Solyc10g008520.2	−1.36	1.43 × 10^−29^	Indole-3-acetic acid-amido synthetase GH3.10
Solyc11g013310.1	−1.91	2.97 × 10^−37^	Auxin transporter-like protein 3
Solyc03g123410.1	−1.96	6.59 × 10^−35^	auxin-binding protein ABP19a
Solyc11g069190.1	−2.00	2.29 × 10^−35^	Auxin response factor
Solyc09g056390.1	−2.13	2.09 × 10^−66^	auxin-induced in root cultures protein 12
Solyc07g041720.1	−2.99	2.45 × 10^−162^	auxin-binding protein ABP19a-like
Auxin (stage 4)			
Solyc07g066560.1	2.29	5.62 × 10^−30^	auxin-responsive protein SAUR71
Solyc06g075690.2	2.14	0	Auxin-regulated protein
Solyc12g014500.1	1.87	2.91 × 10^−13^	indole-3-acetate O-methyltransferase 1-like
Solyc04g081240.2	1.07	1.16 × 10^−6^	Auxin response factor 5
Solyc04g076850.2	−1.14	1.62 × 10^−94^	Auxin-responsive protein
Solyc09g075210.2	−1.35	2.30 × 10^−182^	indole-3-acetic acid-induced protein ARG2
Solyc02g079190.2	−1.64	5.46 × 10^−122^	protein AUXIN SIGNALING F-BOX 2
Solyc06g063060.2	−2.06	3.94 × 10^−21^	Auxin repressed protein
Solyc09g083290.2	−2.79	1.06 × 10^−35^	Auxin-responsive protein
Solyc06g053840.2	−2.79	3.09 × 10^−34^	Auxin-responsive protein
Gibberellins (stage 3)			
Solyc02g070430.2	6.31	2.08 × 10^−24^	gibberellin 2-beta-dioxygenase 1
Solyc12g042500.1	2.63	3.77 × 10^−08^	Gibberellin regulated protein
Solyc07g061730.2	2.32	1.09 × 10^−20^	Gibberellin 2-oxidase
Solyc07g056670.2	1.93	1.71 × 10^−15^	Gibberellin 2-oxidase 2
Solyc01g111080.2	1.59	2.12 × 10^−43^	Gibberellin-regulated protein 1
Solyc03g113910.2	1.42	2.34 × 10^−06^	gibberellin-regulated protein 10
Solyc06g008870.2	1.11	4.70 × 10^−12^	gibberellin receptor GID1B-like
Solyc02g089350.2	−2.50	8.37 × 10^−46^	Gibberellin regulated protein
Solyc03g119910.2	−3.83	7.14 × 10^−15^	Gibberellin 3-beta-dioxygenase 1
Solyc03g116060.2	−5.80	5.10 × 10^−4^	Gibberellin-regulated protein 4
Gibberellins (stage 4)			
Solyc12g042500.1	2.48	2.00 × 10^−4^	Gibberellin regulated protein
Solyc04g078200.2	1.95	2.94 × 10^−27^	gibberellin-regulated family protein precursor
Solyc03g116060.2	1.70	7.30 × 10^−3^	Gibberellin-regulated protein 4
Solyc06g007890.2	1.52	1.56 × 10^−2^	Gibberellin regulated protein
Solyc07g063940.1	1.28	5.31 × 10^−62^	Chitin-inducible gibberellin-responsive protein 1
Solyc01g111080.2	1.15	2.47 × 10^−198^	Gibberellin-regulated protein 1
Solyc07g061720.2	−1.02	8.70 × 10^−4^	Gibberellin 2-oxidase
Solyc11g072310.1	−1.54	1.00 × 10^−2^	Gibberellin 20-oxidase-3
Solyc07g061730.2	−3.96	7.97 × 10^−18^	Gibberellin 2-oxidase
Cytokinin (stage3)			
Solyc04g014990.1	3.46	1.82 × 10^−8^	zeatin O-xylosyltransferase-like
Solyc01g088160.2	2.71	3.54 × 10^−94^	Cytokinin oxidase/dehydrogenase-like
Solyc10g079930.1	2.25	2.63 × 10^−48^	zeatin O-glucosyltransferase-like
Solyc06g062330.1	2.05	5.77 × 10^−19^	zeatin O-glucosyltransferase-like
Solyc04g081290.2	2.00	9.30 × 10^−19^	Cytokinin riboside 5′-monophosphate phosphoribohydrolase
Solyc04g008310.1	1.67	5.45 × 10^−38^	zeatin O-xylosyltransferase-like
Solyc08g062820.2	1.56	7.56 × 10^−31^	Cytokinin riboside 5′-monophosphate phosphoribohydrolase
Solyc12g008900.1	1.74	2.16 × 10^−8^	cytokinin dehydrogenase 3 isoform X1
Solyc05g053400.1	−1.95	1.05 × 10^−18^	zeatin O-xylosyltransferase-like
Solyc11g066670.1	−2.16	1.66 × 10^−56^	zeatin O-glucosyltransferase-like
Cytokinin (stage4)			
Solyc01g088160.2	1.79	5.20 × 10^−18^	Cytokinin oxidase/dehydrogenase-like
Solyc06g075090.2	1.61	1.25 × 10^−6^	Cytokinin riboside 5′-monophosphate phosphoribohydrolase
Solyc06g062330.1	−1.18	1.60 × 10^−3^	zeatin O-glucosyltransferase-like

## Supplementary Materials

The following figures are available online at https://www.mdpi.com/1422-0067/21/16/5914/s1. Table S1: The top five up-/down-regulted DEGs, Table S2: GO terms enriched by DEGs, Table S3: KEGG pathways enriched by DEGs, Table S4: The DEGs related to cell expansion, auxin, gibberellins and cytokinin in *dsp* sepal.
